# Microtubule-binding protein FOR20 promotes microtubule depolymerization and cell migration

**DOI:** 10.1038/celldisc.2017.32

**Published:** 2017-09-05

**Authors:** Sijie Feng, Yinlong Song, Minhong Shen, Shanshan Xie, Wenjing Li, Yi Lu, Yuehong Yang, Guangshuo Ou, Jun Zhou, Fudi Wang, Wei Liu, Xiaoyi Yan, Xin Liang, Tianhua Zhou

**Affiliations:** 1 Department of Cell Biology and Program in Molecular Cell Biology, Zhejiang University School of Medicine, Yuhangtang Road, Hangzhou, Zhejiang, China; 2 Collaborative Innovation Center for Diagnosis and Treatment of Infectious Diseases, Hangzhou, Zhejiang, Hangzhou 310058, China; 3 Tsinghua-Peking Center for Life Sciences, School of Life Sciences, Tsinghua University, Beijing 100084, China; 4 Max-Planck Partner Group, School of Life Sciences, Tsinghua University, Beijing, Tianjing 300073, China; 5 Department of Genetics and Cell Biology, College of Life Sciences, Nankai University, Tianjin, China

**Keywords:** FOR20, microtubules, dynamics, microtubule depolymerization, tubulin sequestering, cell migration

## Abstract

Microtubules are highly dynamic filaments assembled from αβ-tubulin heterodimers and play important roles in many cellular processes, including cell division and migration. Microtubule dynamics is tightly regulated by microtubule-associated proteins (MAPs) that function by binding to microtubules or free tubulin dimers. Here, we report that FOR20 (FOP-related protein of 20 kDa), a conserved protein critical for ciliogenesis and cell cycle progression, is a previously uncharacterized MAP that facilitates microtubule depolymerization and promotes cell migration. FOR20 not only directly binds to microtubules but also regulates microtubule dynamics *in vitro* by decreasing the microtubule growth rate and increasing the depolymerization rate and catastrophe frequency. In the *in vitro* microtubule dynamics assays, FOR20 appears to preferentially interact with free tubulin dimers over microtubules. Depletion of FOR20 inhibits microtubule depolymerization and promotes microtubule regrowth after the nocodazole treatment in HeLa cells. In addition, FOR20 knockdown significantly inhibits both individual and collective migration of mammalian cells. Taken together, these data suggest that FOR20 functions as a MAP to promote microtubule depolymerization and cell migration.

## Introduction

Microtubules have pivotal roles in fundamental cellular processes, such as cell division, intracellular transport and cell migration [1, 2]. They are highly dynamic filaments assembled from αβ-tubulin heterodimers [3–6]. During microtubule dynamics, microtubules undergo periods of growth and shrinkage with transitions between two phases, called catastrophe (from polymerization to depolymerization) and rescue (from depolymerization to polymerization) [3–6]. In general, microtubule dynamic instability can be described by four parameters: the rate of growth, rate of shrinkage, catastrophe frequency and the rescue frequency [6].

The microtubule dynamics is regulated by the hydrolysis of β-tubulin-bound GTP [4]. GTP bound to β-tubulin makes microtubules more prone to polymerization, whereas microtubules with GDP bound to β-tubulin tend to depolymerize [4]. A non-hydrolyzable GTP analog, guanosine-5′- [(α,β)-methylene] triphosphate (GMPCPP), binds to the tubulin exchangeable nucleotide binding site and stabilizes microtubule [6–8]. The antitumor drug taxol also interacts with and stabilizes microtubules by preventing microtubule depolymerization even in the absence of exogenous GTP [8–10]. In cells, microtubule dynamics is tightly regulated (that is, stabilizing or destabilizing) by microtubule-associated proteins (MAPs) that act by binding to the microtubule lattice or free tubulin dimers [11–13].

The centrosome is the main microtubule-organizing center in most animal cells [14–16]. Many centrosome-associated proteins are found to regulate microtubule dynamics [17, 18]. Recently, a conserved centrosomal protein, FOR20 (FOP-related protein of 20 kDa) has been reported to play essential roles in ciliogenesis [19, 20]. In the multiciliated unicellular organism *Paramecium*, PtFOR20p (the ortholog of human FOR20) is recruited in the early course of basal body biogenesis to build the transition zone and is required for basal body docking at the cell surface [20]. In mammalian cells, depletion of FOR20 significantly decreases the percentage of ciliated cells and the lengths of their cilia [19]. In addition, our group has found that FOR20 is critical for S-phase progression by recruiting polo-like kinase 1 to centrosomes [21]. However, little is known about the role of the centrosomal protein FOR20 in microtubule dynamics.

Here, we report for the first time that FOR20 is able to directly promote microtubule depolymerization and cell migration. FOR20 interacts with microtubules and regulates microtubule dynamics by decreasing the microtubule growth rate, increasing the depolymerization rate and catastrophe frequency *in vitro*. In mammalian cells, knockdown of FOR20 significantly inhibits microtubule depolymerization and cell migration. These results indicate that FOR20 functions as a previously unrecognized MAP to facilitate the microtubule destabilization, which is required for cell migration.

## Results

### FOR20 interacts with microtubules

**Figure 1:**
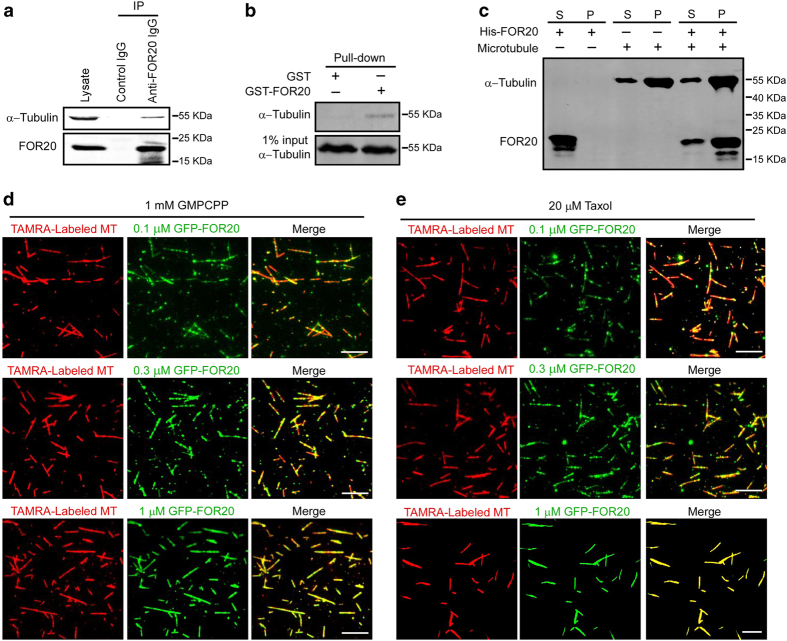
FOR20 interacts with microtubules. (**a**) Lysates of HeLa cells were subjected to immunoprecipitation with control IgG or anti-FOR20 antibodies, followed by western blotting with the indicated antibodies. (**b**) Purified GST or GST-FOR20 protein was incubated with lysates of HeLa cells and processed for Western analysis with anti-α-tubulin antibody. 1% of total input is shown. (**c**) Taxol-stabilized microtubules were incubated with purified His-FOR20 and sedimented by ultracentrifugation. The supernatant (S) and pellet (P) fractions were analyzed by immunoblotting with anti-α-tubulin and FOR20 antibodies. (**d** and **e**) Microtubules polymerized with TAMRA (tetramethyl rhodamine)-labeled tubulin and unlabeled tubulin (1: 9) in the presence of GMPCPP (**d**) or taxol (**e**) were incubated with different concentrations of purified GFP-FOR20 protein on a cover glass surface coated with anti-TAMRA antibody, and then imaged using TIRF microscopy. Scale bar, 5 μm.

To explore the roles of FOR20 in microtubule dynamics, we first examined whether the FOR20 protein is associated with tubulin in cells. Our immunoprecipitation and GST pull-down experiments with cell lysates showed that FOR20 interacted with tubulin (Figure 1a and b). Then we checked if the purified FOR20 has a direct interaction with microtubules. In the microtubule cosedimentation assay, the majority of purified FOR20 was pelleted down with taxol-stabilized microtubules, whereas FOR20 was remained in the supernatant fraction without microtubules in the control group (Figure 1c). To confirm the interaction between FOR20 and microtubules, we incubated the purified GFP-FOR20 with GMPCPP-stabilized microtubules that were immobilized on the surface of coverslips. By using total internal reflection fluorescence (TIRF) microscopy, we observed the clear co-localization of GFP-FOR20 and microtubules (Figure 1d). GFP-FOR20 was also found to interact with taxol-stabilized microtubules (Figure 1e). The similar binding behavior of FOR20 on GMPCPP-stabilized and taxol-stabilized microtubules suggest that the purified FOR20 has no preference for the GTP (as shown by GMPCPP-stabilized microtubules) or GDP (as shown by taxol-stabilized microtubules) status of microtubules. Collectively, these data indicated that FOR20 is a microtubule-binding protein.

### FOR20 facilitates microtubule destabilization *in vitro*

**Figure 2:**
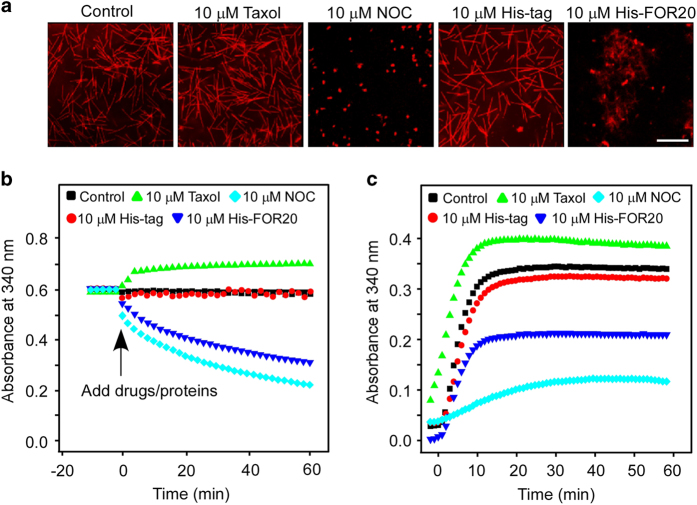
FOR20 inhibits microtubule polymerization *in vitro.* (**a**) Microtubules were assembled with rhodamine-labeled and unlabeled tubulin (1:9) in the presence of taxol (20 μm) and GTP (1 mm). The assembled microtubules were then added by the indicated purified protein or chemicals, and processed for confocal microscopy. Scale bar, 5 μm. His-tag, a peptide of six histidine residues; NOC, nocodazole. (**b**) Microtubules assembled with tubulin (17 μm) in the presence of taxol (20 μm) and GTP (1 mm) were added by the indicated purified protein or chemicals, and then measured by light scattering at 340 nm wavelength with spectrophotometer. Data are analyzed by the GraphPad Software (Inc. La Jolla, CA, USA). (**c**) Tubulin dimers (17 μm) were mixed with the indicated protein or chemicals in the presence of taxol (20 μM) and GTP (1 mM) and subjected to spectrophotometer analysis. Data are plotted using the GraphPad Prism 5 program.

Since FOR20 interacts with microtubules, we further investigated the role of FOR20 in microtubule dynamics. We employed an *in vitro* microtubule assembly assay with taxol and GTP and found that purified His-FOR20 induced microtubule depolymerization (Figure 2a), resembling the effect of nocodazole (a microtubule-destabilizing agent) treatment. Subsequently, the microtubule turbidity assay was used to analyze the effect of FOR20 on the kinetics of microtubule assembly and disassembly. The results showed that FOR20 caused the depolymerization of the pre-assembled microtubules and inhibited microtubule polymerization *in vitro* (Figure 2b and c), implying that FOR20 may be a microtubule destabilizer.

**Figure 3:**
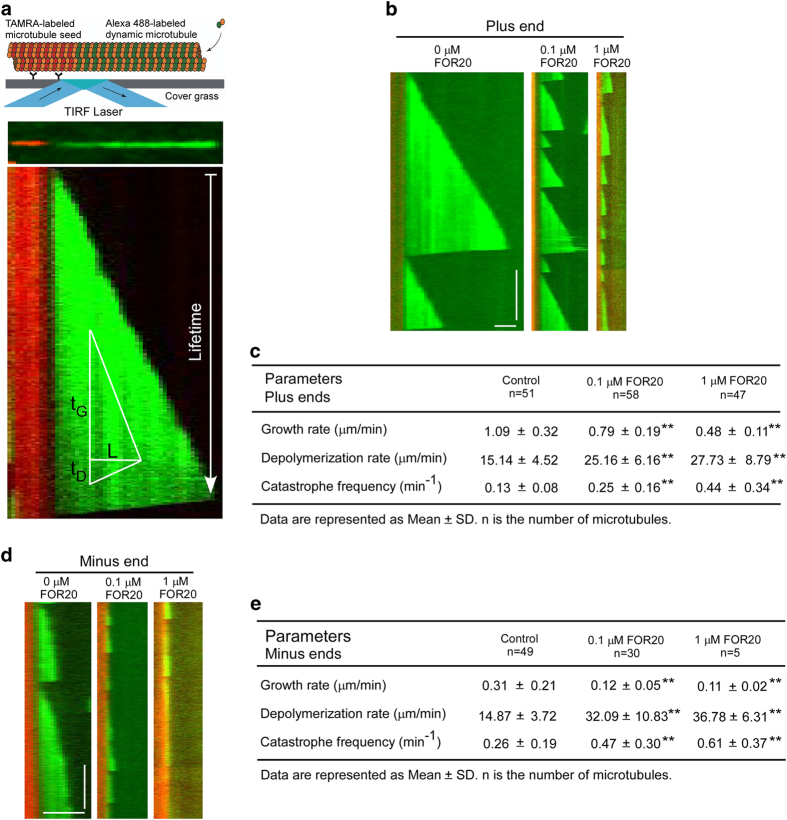
FOR20 decreases the microtubule growth rate and increases the depolymerization rate and catastrophe frequency. (**a**) The schematic of *in vitro* microtubule dynamics assay depicts microtubules grown from a TAMRA-labeled microtubule seed that was immobilized on a cover glass surface by anti-TAMRA antibody. Tubulin polymerization is imaged by TIRF microscopy. In the kymograph, the vertical distance indicates time and the horizontal distance represents microtubule length. Microtubule length/time is the microtubule growth rate (L/t_G_) or depolymerization rate (L/t_D_). Lifetime is t_G_, and the catastrophe frequency is 1/t_G_. (**b**–**e**) 10% Alexa 488-labeled microtubules (12 μM free tubulin dimers) were grown from 10% TAMRA-labeled microtubule seeds (red) stabilized by GMPCPP (1 mM) in the presence of different concentrations of FOR20 on a cover glass surface coated with anti-TAMRA antibody, and then detected by TIRF microscopy. Kymograph depicts dynamic microtubules from plus (b and c) and minus (d and e) ends during microtubule growing and shrinking. The growing microtubule tip position was measured by ImageJ software (Fiji) to evaluate kinetic parameters of microtubule dynamics. **P<0.01, student’s *t*-test. Vertical bar, 5 min; horizontal bar, 5 μm. Also see Supplementary Movie S1, Supplementary Movie S2, Supplementary Movie S3.

To further understand how FOR20 promotes microtubule destabilization at the molecular level, we investigated the effect of the purified FOR20 on microtubule dynamics with an *in vitro* microtubule dynamics assay. In this experiment, we used 10% Alexa 488-labeled free tubulin dimers to polymerize dynamic microtubules from the GMPCPP-stabilized microtubule seeds (Figure 3a). The dynamic behavior of microtubules was recorded by TIRF microscopy and analyzed by ImageJ software (Fiji) [22]. The kymograph analysis based on the single microtubule plus end dynamics showed that the purified FOR20 decreased the microtubule growth rate, and increased the depolymerization rate and catastrophe frequency (Figure 3b and c and Supplementary Movie S1, Supplementary Movie S2, Supplementary Movie S3). Similar effects were also observed on the microtubule minus ends (Figure 3d and e and Supplementary Movie S1, Supplementary Movie S2, Supplementary Movie S3). The inhibitory roles of FOR20 in microtubule dynamics were dose-dependent (Figure 3c and e). Taken together, these results indicate that FOR20 facilitates microtubule destabilization.

### FOR20 associates with free tubulin dimers

**Figure 4:**
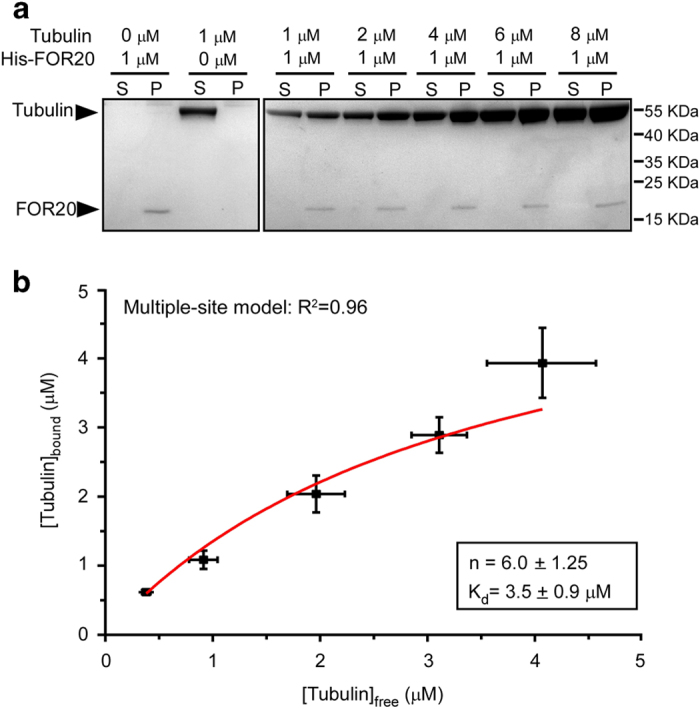
FOR20 associates with free tubulin dimers *in vitro*. (**a**) Different concentrations of free tubulin dimers were incubated with purified His-FOR20 (1 μm) and then added by Ni-NTP beads. After centrifugation, the supernatant (S) and pellet (P) fractions were analyzed by gel electrophoresis with Coomassie blue staining. (**b**) The intensities of bands were quantified by ImageJ. The FOR20-bound tubulin dimer ([Tubulin]_bound_) was plotted against free tubulin dimer ([Tubulin]_free_). The results were fit to the bimolecular binding curve to obtain the apparent *K*_d_ (the disassociation constant) and *n* (the number of the tubulin-binding sites of FOR20). Data are expressed as mean±s.d (more than three independent experiments).

To determine the mechanism how FOR20 regulates microtubule destabilization, we tried to test whether FOR20 directly binds to free tubulin dimers and found that His-FOR20 was able to pull down purified tubulin dimers *in vitro* (Figure 4a), in agreement with the interaction between FOR20 and tubulin in cells (Figure 1a and b). To further measure the binding stoichiometry of free tubulin dimers to FOR20, we used a fixed concentration of purified FOR20 (1 μm) and titrated a series of tubulin concentrations. When [Tubulin]_total_ ([Tubulin]_bound_+ [Tubulin]_free_) was 1, 2, 4, 6 and 8 μm, the FOR20-bound fraction of tubulin dimer, that is [Tubulin]_bound_, was 0.6, 1.1, 2.4, 2.9 and 3.9 μm, respectively (Figure 4b). We fitted the data to the biochemical model that assumes FOR20 with multiple, identical and independent binding sites for tubulin dimers [23]. Under this condition, one FOR20 molecule appeared to associate with about five to seven tubulin dimers (*n*=6.0±1.25) with a *K*_d_ of 3.5±0.9 μm (Figure 4b). These results reveal that FOR20 directly binds to several free tubulin dimers, implying that the tubulin sequestering mechanism may be involved in FOR20-mediated microtubule destabilization.

### FOR20 has no end-tracking function

**Figure 5:**
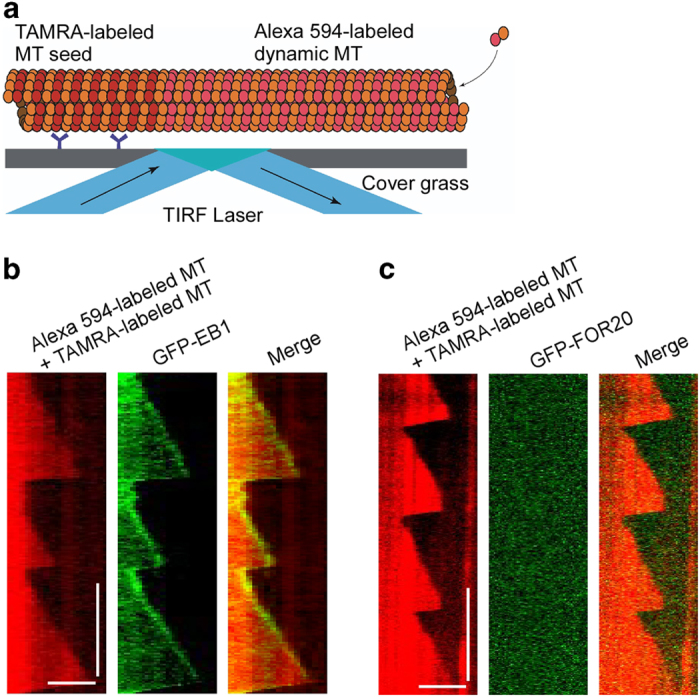
FOR20 has no obvious microtubule end-tracking function. (**a**) The schematic representation of *in vitro* microtubule dynamics assay describes microtubules grown from a TAMRA-labeled microtubule seed that was immobilized on a cover glass surface by anti-TAMRA antibody. Tubulin polymerization is imaged by TIRF microscopy. (**b** and **c**) Microtubules were polymerized with Alexa 594-labeled tubulin by extension of TAMRA-labeled microtubule seeds. Purified GFP-EB1 (30 nm) (**b**) or GFP-FOR20 (150 nm) (**c**) was added to detect the corresponding end-tracking function by TIRF microscopy. Kymograph depicts microtubule dynamics during microtubule growing and shrinking. MT, microtubule. Vertical bar, 5 min; horizontal bar, 5 μm. Also seeSupplementary Movie S3, Supplementary Movie S4.

To explore whether FOR20 regulates microtubule dynamics through the interaction between FOR20 and the microtubule ends, we used an *in vitro* microtubule dynamics assay with 10% TAMRA-labeled microtubule seeds and 10% Alexa 594-labeled tubulin dimers (Figure 5a). In this assay, both the microtubule seeds and the dynamic microtubules were excited using the 561 nm laser. Since the inhibitory effects of GFP-FOR20 were similar to those of the non-tagged FOR20 (Supplementary Figure S1 and Supplementary Movie S6, Supplementary Movie S7), we used GFP-FOR20 in our experiments for the TIRF recording. The results showed that in contrast to the microtubule plus-end-tracking protein EB1, GFP-FOR20 did not have an obvious end-tracking behavior (Figure 5b and c and Supplementary Movie S3, Supplementary Movie S4), which is consistent with our previous observation that GFP-FOR20 had no preference for the GTP or GDP status of tubulin in the microtubule lattice (Figure 1d and e).

### FOR20 destabilizes microtubules in mammalian cells

**Figure 6:**
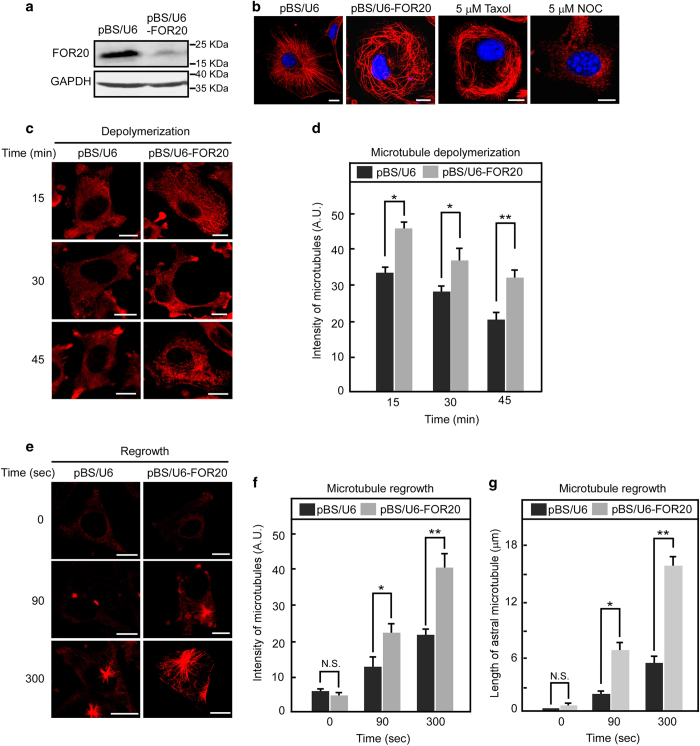
Depletion of FOR20 stabilizes microtubules in mammalian cells. (**a**) HeLa cells transfected with pBS/U6 or pBS/U6-FOR20 plasmid were lysed and subjected to western analysis. GAPDH was used as an internal control. (**b**) The cells with the indicated treatments were used for immunofluoresence analysis with anti-α-tubulin antibody. DNA was visualized with DAPI. Scale bar, 10 μm. (**c** and **d**) HeLa cells were treated with nocodazole (5 μm) for the indicated times and processed for immunostaining with anti-α-tubulin antibody (**c**). The intensity of microtubules was measured by MetaMorph software (Molecular Devices) and data are presented as mean±s.d. (**d**). (**e**–**g**) HeLa cells treated with nocodazole (5 μm) for 3 h and were washed out to allow microtubule regrowth for the indicated times and processed for immunofluoresence (**e**). The intensity (**f**) and astral length (**g**) of microtubules were evaluated by MetaMorph software. The mean fluorescence intensities of 30 cells in each group were determined, and data are expressed as mean±s.d. Scale bar, 10 μm. N.S., not significant (*P*>0.05); *P<0.05 and **P<0.01, student’s *t*-test.

Since FOR20 has an inhibitory effect on microtubule dynamics *in vitro*, we determined the influence of FOR20 on the dynamics of cellular microtubules. We used a vector-based RNAi to deplete the endogenous FOR20 and found that the protein level of FOR20 was efficiently reduced in HeLa cells transfected with the pBS/U6-FOR20 plasmid (Figure 6a). Immunofluorescence analysis of FOR20-depleted cells showed the formation of thickened microtubule bundles that encircled the nucleus, resembling the cellular phenotype caused by taxol treatment (Figure 6b). Furthermore, we investigated cellular microtubule dynamics in cells treated with nocodazole and FOR20 depletion, and discovered that knockdown of FOR20 significantly retarded microtubule depolymerization induced by nocodazole (Figure 6c and d). During the microtubule regrowth after transient treatment with nocodazole, FOR20 depletion significantly promoted microtubule assembly (Figure 6e–g). Collectively, these data suggest that FOR20 acts as a negative regulator of microtubule polymerization in mammalian cells, which is consistent with our observations *in vitro*.

### FOR20 is essential for cell migration

**Figure 7:**
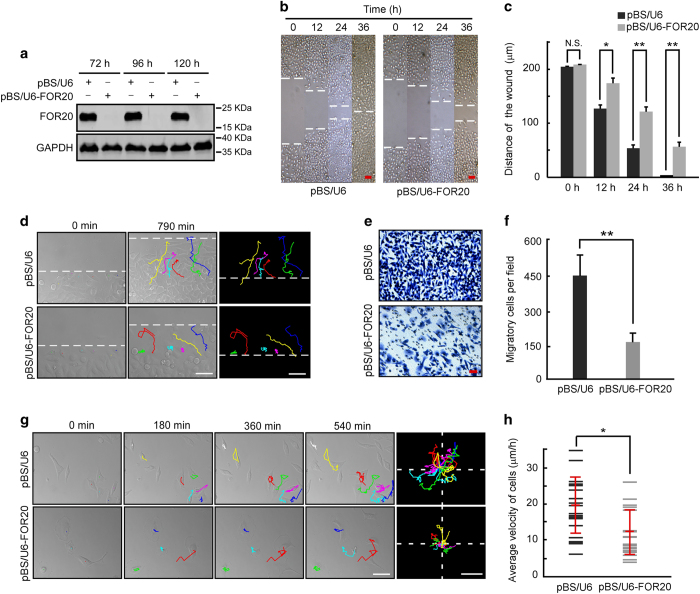
FOR20 knockdown inhibits cell migration. HeLa cells were transfected with either pBS/U6 or pBS/U6-FOR20 plasmid for different durations and subjected to the following assays. (**a**) Western blotting revealed the efficiency of FOR20 depletion in HeLa cells. GAPDH was used as an internal control. (**b** and **c**) The wound healing assay displayed the migration of the control or FOR20-depleted cells. Dashed lines indicate the wound edges (**b**). Scale bar, 50 μm. Distance of the wound was measured by ImageJ software and data are presented as mean±s.d. (**c**). (**d**) HeLa cells were transfected with the indicated plasmids and processed for wound healing assays. The cell migration paths present at the wound edge were traced using ImageJ software. Scale bar, 50 μm. (**e** and **f**) Transwell analysis exhibited the migration of the control or FOR20-depleted cells (**e**). Scale bar, 20 μm. Quantitative data of randomly selected fields (*n*>3) are expressed as mean±s.d. (**f**). (**g** and **h**) The migration tracks of individual cells transfected with the indicated plasmids were traced by ImageJ software. The migration paths were consolidated and re-plotted from the origin. Quantitative data of average velocity are represented as mean±s.d (red horizontal bars). Scale bar, 50 μm. N.S., not significant (*P*>0.05); *P<0.05 and **P<0.01, student’s *t*-test.

Given that microtubule dynamics is required for cell motility [1, 4], we tested if FOR20 has a role in cell migration. Our wound healing assays showed that cells depleted of FOR20 were significantly less motile than the control cells (Figure 7a–c). Tracing the migratory path of cells at the wound edge displayed that the trajectories of FOR20-depleted cells had kinks and bends with reduced wound closure, whereas most control cells were directionally oriented towards the wound (Figure 7d and Supplementary Movie S7, Supplementary Movie S8). To investigate the effect of FOR20 on the directional migration induced by external stimuli, we employed a chemotaxis assay with a modified Boyden chamber and found that the migration of FOR20-depleted cells was significantly reduced compared to that of the control cells (Figure 7e and f). Furthermore, we examined the migratory behavior of FOR20 knockdown cells during random migration. Tracing the cell migration path revealed that cells depleted of FOR20 exhibited a non-directional phenotype with low velocity and extended membrane protrusions around the cell periphery during movement (Figure 7g and h and Supplementary Movie S10, Supplementary Movie S11). On the other hand, the control cells displayed a classic polarized phenotype represented by the formation of a dominant lamella at the front and a narrow trailing edge at the rear. More importantly, the less motile phenotype of FOR20-depleted cells was significantly reversed by ectopic expression of GFP-FOR20 (Supplementary Figure S2). Thus, these results indicate that FOR20 is critical for both individual and collective migration of mammalian cells.

## Discussion

**Figure 8:**
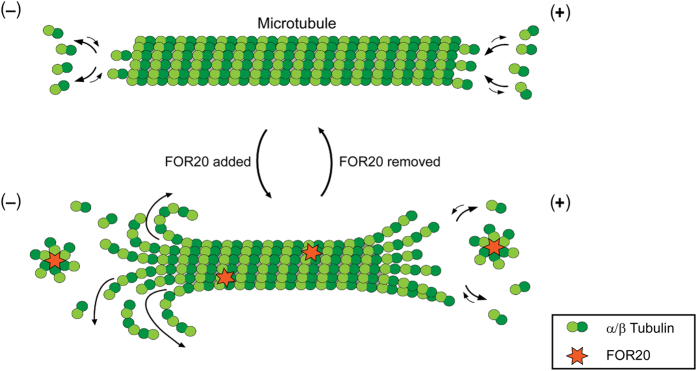
A working model for the regulation of microtubule dynamics by FOR20. Microtubules are dynamic structures due to the assembly and disassembly at plus and minus ends. FOR20 may form a complex with free tubulin dimers to sequester them away from microtubules at both ends. It is still not clear whether the interaction between FOR20 and the microtubule lattice is involved in the regulation of microtubule dynamics by FOR20.

In this study, we provide evidence that FOR20 is a microtubule-binding protein that promotes microtubule depolymerization and inhibits microtubule polymerization (Figure 8), which is essential for cell migration. However, the molecular mechanism of microtubule dynamics regulated by FOR20 still remains elusive. On the basis of biochemical nature of microtubule polymerization [11, 12], we considered three possible working mechanisms of FOR20 in the regulation of microtubule dynamics at the molecular level: (1) the tip binding mechanism that is based on the binding of FOR20 to microtubule ends; (2) the tubulin sequestering mechanism that is based on the direct interaction between FOR20 and free tubulin dimers; (3) the microtubule lattice binding mechanism that is based on the interaction between FOR20 and the microtubule lattice. In our *in vitro* dynamics assays, we found that FOR20 had no obvious end-tracking function (Figure 5), implying that the tip binding mechanism may not contribute to the regulation of microtubule dynamics by FOR20.

In general, the effect of a tubulin sequester is considered as reducing the concentration of free tubulin dimers available for microtubule polymerization [13, 24], indicating that addition of a tubulin sequester decreases the microtubule growth rate. In our microtubule dynamics assays, we observed that addition of 0.1 μm FOR20 was able to reduce the microtubule growth rate by ~27% (Figure 3c). It is equivalent to reducing the concentration of tubulin dimers by more than 1 μm [25]. In other words, this means that one FOR20 molecule needs to sequester more than 10 tubulin dimers to account for the inhibitory effects, which is inconsistent with our observation that one FOR20 molecule binds to ~5–7 free tubulin dimers (Figure 4b). Therefore, the conventional tubulin sequestering mechanism alone cannot fully account for the reduction in the microtubule growth rate to this extent.

More importantly, microtubule depolymerization is usually independent of the concentration of free tubulin dimers because it entails the disassembly of tubulin from existing microtubules [6, 26]. Here, our results showed that FOR20 obviously increased the microtubule depolymerization rate (Figure 3c). Taken together, these data suggest that the tubulin sequestering mechanism may contribute to the inhibitory roles of FOR20 in microtubule formation, but not be the only molecular mechanism underlying how FOR20 destabilizes microtubules.

It is interesting that we found the strong decoration of chemically stabilized microtubules by GFP-FOR20 (Figure 1d and e) with the lack of localization of the same protein on the dynamic microtubules and the GMPCPP seeds in our dynamic assays (Supplementary Figure S1). One possible interpretation is that most of GFP-FOR20 is occupied by free tubulin dimers, but not by the microtubule lattice. In our microtubule dynamics assays (Supplementary Figure S1), microtubules were polymerized with 12 μm Alexa 594-labeled tubulin by extension of GMPCPP-stabilized TAMRA-labeled microtubule seeds, and only 1 μm purified GFP-FOR20 was added. On the basis of the number and the average lengths of microtubules in our microtubule dynamic experiments, we estimated that the number of free tubulin dimers is ~6 800 times compared to that of immobile tubulin dimers in microtubules attached on the coverslip surface (see the detailed calculation in Materials and Methods). Even if GFP-FOR20 has a similar affinity to the polymerized microtubules and free tubulin dimers, most of GFP-FOR20 would bind to free tubulin dimers. Thus, these results imply that the lattice binding mechanism may not be the main mechanism for the inhibitory regulation of FOR20 in our *in vitro* microtubule dynamics assays. Future studies on the regulation of microtubule dynamics by FOR20 in molecular detail are clearly needed.

Recent studies have reported that a number of MAPs stabilize microtubules, whereas relatively few MAPs destabilize microtubules, such as oncoprotein18/stathmin [12, 13], *Xenopus* kinesin catastrophe modulator-1 [27] and katanin family members [28]. Among them, stathmin has similar effects on microtubule dynamics as FOR20, including decreasing the microtubule growth rate and increasing the catastrophe frequency [29, 30]. Intriguingly, a similar mystery surrounding the inhibitory roles of FOR20 is also found in regards to stathmin. Stathmin interacts with two free tubulin dimers [30, 31], but has no clear binding to microtubules [13, 32]. This low molar ratio of stathmin to tubulin dimers in microtubule dynamics assays also cannot account for the strong inhibition of microtubule polymerization by stathmin [12], implying that, as in the case of FOR20, stathmin may not act simply by sequestering free tubulin dimers.

Cell migration plays essential roles in many physiological and pathological processes including embryonic development, wound healing and metastasis [33, 34]. Migration is a polarized cellular process that involves the repetition of four basic steps: protrusion, adhesion, contraction and retraction [1]. Dynamic microtubules have been demonstrated to participate in almost all essential events leading to cell migration [1, 4]. Microtubule dynamics is required for generating an asymmetrical microtubule array and maintaining cell shape [35]. Microtubules also play a critical role in cell protrusion and migration by entering into lamellipodia, consequently pushing the plasma membrane at the leading edge [36, 37]. In this report, we found that FOR20-depleted cells exhibited non-directional phenotypes with low velocities and extended membrane protrusions around the cell periphery during both individual and collective migration (Figure 7). These results suggest the essential roles of FOR20 in cell migration, likely through modulating microtubule dynamics.

Taken all together, here we report the previously undescribed roles of FOR20 in facilitating microtubule depolymerization and cell migration, which expands our understanding of the functions of FOR20.

## Materials and methods

### Cell culture, RNAi and transfection

HeLa cells were cultured in DMEM (Corning, Tewksbury, MA, USA) containing 10% serum (Gibco, Waltham, MA, USA) at 37 °C in 5% CO_2_. Oligos corresponding to the following sequences were synthesized and cloned into the pBS/U6 vector for FOR20 knockdown or control RNAi: 5′- AGGTAGAGGAGAAGTAAAT-3′ for FOR20 RNAi (pBS/U6-FOR20), and 5′- UUCUCCGAACGUGUCACGU-3′ for control RNAi (pBS/U6) [21]. These vectors were transfected into cells with the Lipofectamine 2000 reagent (Invitrogen, Waltham, MA, USA) according to the manufacturer’s instructions.

### Western blotting

Cell lysates or microtubule pellets were subjected to western analysis with anti-FOR20, α-tubulin or GAPDH antibodies (Sigma, St Louis, MO, USA). The blots were probed with either Alexa Fluor 680 or IRDye 800-conjugated secondary antibodies. The Alexa Fluor 680 or IRDye 800CW activity was detected by the Odyssey system (LI-COR Biosciences, Lincoln, NE, USA).

### Protein expression and purification

To generate purified His-FOR20, GFP-FOR20 or GST-FOR20 protein, full-length FOR20 and GFP-FOR20 were subcloned into the pET28a vector containing an N-terminal histidine tag (His) or pEGX-5X vector containing an N-terminal glutathione S-transferase tag (GST). The plasmids were transformed into *Escherichia coli* BL21. Single clone containing His-FOR20, His-GFP-FOR20 or GST-FOR20 was picked and the bacteria were incubated for 16 h at 16 °C after IPTG induction. These bacteria were harvested and the lysates were incubated with nickel-coated or glutathione-agarose beads for 2 h at 4 °C in the presence of protease inhibitors for purification. His-tag of FOR20 and GFP-FOR20 were removed by thrombin. FOR20 and GFP-FOR20 were then dialyzed using the microtubule dynamics assay buffer BRB80 (80 mm PIPES, pH 6.8, 1 mm MgCl_2_, 1 mm EGTA) and supplemented with 20% glycerol. GST-FOR20 was dialyzed using PBS (phosphate buffer saline) with 20% glycerol. These purified proteins were snap-frozen in liquid nitrogen and stored in −80 °C.

### GST pull down assay

GST pull down assay was performed as described previously [21]. In brief, purified GST or GST-FOR20 protein was incubated with lysates of HeLa cells in TBSN (20 mm Tris, pH 8.0, 150 mm NaCl, 0.5% NP-40, 5 mm EGTA, 1.5 mm EDTA, 0.5 mm Na_3_VO_4_, 20 mm *p*-nitrophenyl phosphate) containing protease inhibitors at 4 °C for 2 h. Then, GST and GST-FOR20 were adsorbed to glutathione-agarose beads for additional 2 h. The beads were collected by centrifugation and washed five times. The bound proteins were eluted and detected by western blotting with anti-α-tubulin and FOR20 antibodies.

### Microtubule cosedimentation assay

To examine the interaction between His-FOR20 and microtubules *in vitro*, microtubules were assembled from *porine* brain tubulin dimers at 37 °C in the presence of 1 mm GTP and 20 μm taxol. Taxol-stabilized microtubules were incubated with 10 μm His-FOR20 and 20 μm taxol, and then centrifuged at 100 000 *g* for 20 min at 25 °C. The supernatant and pellet fractions were collected separately and analyzed by western blotting with anti-α-tubulin and FOR20 antibodies.

### Microtubule assembly

For the microtubule polymerization by GMPCPP [38], short microtubule seeds were prepared by incubating 8 μM *porcine* brain tubulin mix containing 10% TAMRA-labeled tubulin with 1 mm GMPCPP (the slowly hydrolyzing GTP analog guanosine-5′- [(α,β)-methylene] triphosphate) and 4 mm MgCl_2_. After incubating on ice for 5 min, the mixture was polymerized in a 37 °C water bath for 2 h in the dark. Then, 400 μl of warm BRB80 buffer was added to stop the reaction. The mixture was ultra-centrifuged with warm spin. The supernatant was removed and the final microtubule seeds were resuspended in warm BRB80. For the microtubule dynamics assays and the co-localization assays of GFP-FOR20 and microtubules, these GMPCPP-stabilized microtubule seeds were attached to a cover glass surface coated with anti-TAMRA antibody and analyzed by TIRF microscopy.

For the microtubule polymerization by taxol, short microtubule seeds were prepared by incubating 32 μm *porcine* brain tubulin mix containing 10% TAMRA-labeled tubulin with 20 μm taxol, 1 mm GTP, 4 mm MgCl_2_ and 4% DMSO. After incubation on ice for 5 min, the mixture was polymerized in a 37 °C water bath for more than 30 min in the dark. Then, 400 μl of warm BRB80 buffer (containing 20 μm taxol) was added to stop the reaction. The sample was subjected to airfuge centrifugation to collect microtubule seeds in the taxol-BRB80 buffer. For *in vitro* microtubule assembly assays, these taxol-stabilized microtubule seeds were centrifuged onto coverslips, added by the indicated purified proteins or chemicals, and immediately subjected to confocal microscopy (LSM510, Zeiss, Oberkochen, Germany). For the co-localization assays of GFP-FOR20 and microtubules, these short microtubule seeds were attached to a cover glass surface coated with anti-TAMRA antibody and then analyzed by TIRF microscopy.

### Tubulin turbidity assay

The microtubule polymerization or depolymerization was monitored by measuring the changes in absorbance (340 nm) by a spectrophotometer as described previously [39, 40]. In brief, all of the components of the reactions in the 96-well plates were incubated at 37 °C for 1 min. After gentle mixing, the reaction mixtures were immediately determined by a spectrophotometer at 37 °C.

### Microtubule dynamics assay

10% Alexa 488-labeled tubulin was assembled onto 10% TAMRA-labeled microtubule seeds and imaged by TIRF microscopy as previously described [41, 42]. Briefly, to keep the microtubules within the excitation field, the microtubule seeds polymerized by GMPCPP were attached to a cover glass surface coated with anti-TAMRA antibody. Microtubules were polymerized with Alexa 488-labeled and unlabeled tubulin (1:9) from the GMPCPP-stabilized and TAMRA-labeled microtubule seeds in the presence of 1 mm GTP and the indicated concentrations of FOR20. The mixture was incubated in image buffer for 5 min at 35 °C and then introduced into the imaging chamber in the IX83HB constant temperature incubator (Olympus IX83-ZDC microscope, Shinjuku, Tokyo, Japan) to observe microtubule dynamics. The image buffer consisted of BRB80 supplemented with 2 mm GTP, 80 mm d-glucose, 0.4 mg ml^−1^ glucose oxidase, 0.2 mg ml^−1^ catalase, 0.8 mg ml^−1^ casein, 1% β-Mercaptoethanol, 0.001% Tween-20. Images were collected with an Andor 897 Ultra EMCCD (Andor, Belfast, UK) using a 100X/1.45 NA TIRF objective. Images with 100 ms exposure time were recorded every 2.5 s, 488 and 561 nm lasers were used to excite the fluorescent labels.

### Kymograph analysis

The dynamic microtubule tip position (plus or minus end) during growing and shrinking was measured by ImageJ software (Fiji, NIH) [22] to evaluate kinetic parameters of microtubule dynamics. In the kymograph, the vertical distance represents time and the horizontal distance indicates the growing microtubule length. The ratio between the microtubule length and the growth/depolymerization time is the microtubule growth rate (L/t_G_) or depolymerization rate (L/t_D_). Lifetime (t_G_) is the period of dynamic microtubule lattice growth from microtubule seeds to the occurrence of catastrophe. The catastrophe frequency (1/t_G_) is the reciprocal of the catastrophe time.

### Protein binding assay

Purified His-FOR20 (1 μm) and different concentrations of tubulin dimers (1–8 μm) were mixed and incubated on ice for 1 h. Ni-NTP beads were added to the mixture, and incubated for another 30 min on ice. The mixture was then centrifuged with 3 000 r.p.m. for 2 min at 4 °C. The supernatants and pellets were collected for gel electrophoresis with Coomassie blue staining. The intensities of the protein bands were measured by ImageJ as an estimation of the protein amounts. The tubulin-binding sites of FOR20 were assumed identical and independent, the data were fitted to a biochemical binding equation: [Tubulin]_bound_=*n*× [His-FOR20]× [Tubulin]_free_/(*K*_d_+ [Tubulin]_free_) [43]. *K*_d_ denotes the disassociation constant and *n* denotes the number of the tubulin-binding sites of FOR20. The [Tubulin]_bound_ and [Tubulin]_free_ represent the concentration of His-FOR20-bound tubulin and free tubulin, respectively.

### Immunofluorescence

HeLa cells grown on coverslips were fixed with 4% paraformaldehyde and rinsed three times with PBS. After blocking in 5% BSA (bovine serum albumin), cells were permeabilized with 0.1% Triton X-100 in PBS, then incubated with primary antibodies diluted in 5% BSA in PBS for 2 h. After washing, Cy3-conjugated anti-mouse IgG or FITC-conjugated anti-rabbit IgG secondary antibodies were added to incubate for another 1 h at room temperature. The DNA was stained with DAPI (Sigma). Finally, the coverslips were mounted and analyzed by confocal microscopy (LSM510, Zeiss).

### Cellular microtubule depolymerization and regrowth

For the cellular microtubule depolymerization assay, HeLa cells grown on coverslips were treated with 5 μm nocodazole for the indicated times, and then fixed for immunofluorescence staining. For the cellular microtubule regrowth assay, HeLa cells grown on coverslips were incubated with 5 μm nocodazole for 3 h to depolymerize microtubules, and then carefully washed out to remove nocodazole followed by fixation at the indicated times. All cells were stained with mouse anti-α-tubulin primary antibody and Cy3-conjugated anti-mouse IgG secondary antibody. The coverslips were then mounted and imaged by confocal microscopy (LSM510, Zeiss). The irregular circles for the region of interest in confocal images were drawn along the cell periphery. The intensities of the red signal that represented microtubules within the region of interest were quantified using MetaMorph software (Molecular Devices, Sunnyvale, CA, USA). The mean fluorescence intensities of 30 cells in each group were determined. Data are expressed as mean±s.d. and analyzed by student’s *t*-test.

### Wound healing assay

HeLa cells were transfected with either pBS/U6 or pBS/U6-FOR20. After transfection for 48 h, the cells were trypsinized and reseeded into 30 mm dishes with 10% serum culture medium. When the cells became confluent, the cells were starved for 12 h and scratched with a 20 μl pipette tip to create wounds. Then, the cells were washed several times with PBS to remove floating cells and debris, and cultured with 1% serum culture medium to allow wound healing. The cells were monitored with an Olympus microscope and representative images were taken at the indicated time points. At least three independent pictures were quantified by using imageJ software [44].

### Chemotaxis assay

After transfection with PBS/U6 or PBS/U6-FOR20 for 48 h, HeLa cells were trypsinized and resuspended at a density of 1×10^5^ per ml in the medium with 1% fetal bovine serum. Then, 200 μl of cells were seeded in the upper compartment of Boyden chambers (6.5 mm pore size; Costar), while 600 μl of the medium containing 20% fetal bovine serum was added to the lower compartment. After incubation for 24 h, the upper side of the chamber was wiped with a cotton swab to remove the cells. The migratory cells on the bottom surface of the chamber were fixed with 4% paraformaldehyde for 20 min and stained with Coomassie brilliant blue.

### Time-lapse microscopy

HeLa cells transfected with either pBS/U6-FOR20 or pBS/U6 plasmids were maintained in an incubation chamber (37 °C with 5% CO_2_) equipped with the Zeiss LSM510 confocal microscope platform. Images were taken every 5 min with AIM Image examiner software to monitor cell migration. The acquired image sequences were analyzed by ImageJ software. The cell migration paths were determined as tracks of nuclei [45].

### Estimation of the amount of tubulin dimers in the immobilized microtubule seeds

In our microtubule dynamics assay, each flow cell has a volume of about 7 μl. The area to immobilize the GMPCPP microtubule seeds in each channel is 54 mm^2^. The total length of the GMPCPP microtubule seeds in each field of view (81.92×81.92 μm^2^) was measured as 526±107 μM (mean±s.d.) by using ImageJ software, the total length of microtubules in each flow cell was summed up to 4.23 m (526 μm×54 mm^2^/(81.92×81.92 μm^2^)=4.23 m). Given that the length of each tubulin dimer is 8 nm and the GMPCPP microtubule is assembled by 14 protofilaments [11, 46], there are 1.23×10^−14^ mol tubulin dimers contained in the GMPCPP microtubule (14×4.23 m/8 nm/(6.02×10^23^mol^−1^)=1.23×10^−14^ mol). The free tubulin dimers are 8.4×10^−11^ mol in our dynamic assays in the 7 μl channel (7 μl×12 μm=8.4×10^−11^ mol). Therefore, the amount of free tubulin dimers is about 6 800 times compared to that of immobile tubulin in microtubules attached on the coverslip surface (8.4×10^−11^ mol/(1.23×10^−14^ mol)=6 829).
