# Supplementary material for: Microtubule-binding protein FOR20 promotes microtubule depolymerization and cell migration
Source: Cell Discov. 2017 Sep 5;3:17032–. doi: 10.1038/celldisc.2017.32 (PMC5583970; doi:10.1038/celldisc.2017.32)
Supplement: Supplementary Information [file celldisc201732-s1.pdf]

**Supplementary information**

a

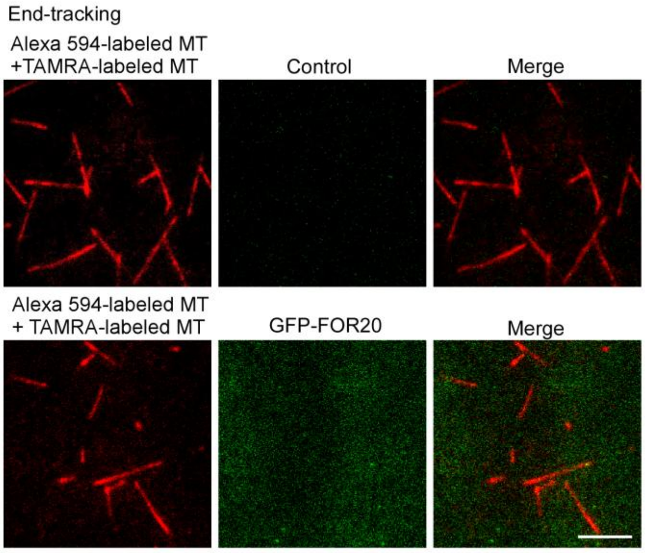


b

The regulation of microtubule dynamics by GFP-FOR20

| Parameters<br>Plus-ends | Control<br>n=36 | 1μM GFP-FOR20<br>n=42 |
|---|---|---|
| Growth rate (μm/min) | 0.87 ± 0.09 | 0.45 ± 0.15** |
| Depolymerization rate (μm/min) | 10.89 ± 2.82 | 18.50 ± 2.54** |
| Catastrophe frequency ($min^{-1}$) | 0.15 ± 0.10 | 0.41 ± 0.25** |

Data are represented as Mean±SD. n is the number of microtubules.

**Supplementary Figure S1. GFP-FOR20 decreases the microtubule growth rate and increases the depolymerization rate and catastrophe frequency**. (**a** and **b**) 10% Alexa 594-labeled microtubules were grown from 10% TAMRA-labeled microtubule seeds stabilized by GMPCPP (1 mM) in the presence of 1 μM GFP-FOR20 on a cover glass surface coated with anti-TAMRA antibody, and then detected by TIRF microscopy. The growing microtubule tip position was measured by ImageJ software to evaluate kinetic parameters of microtubule dynamics. MT, microtubule. **$P < 0.01$, student's *t*-test. Bar, 5 μm. Also see Supplementary Movie S6 and S7.

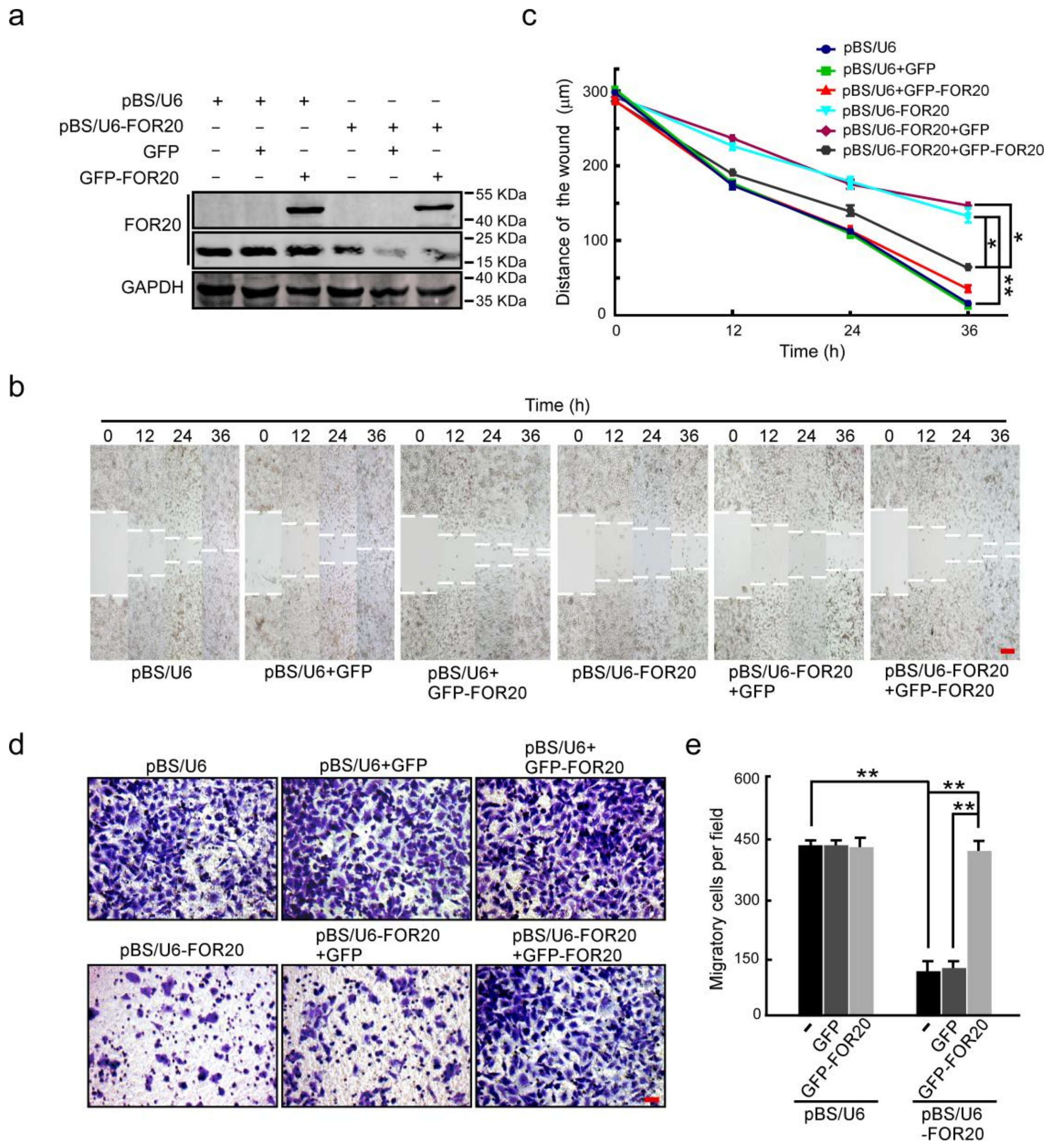


**Supplementary Figure S2. Depletion of FOR20 inhibits cell migration.** HeLa cells were transfected with the indicated plasmids and then subjected to the following assays. (**a**) Western blotting revealed the expression level of endogenous FOR20 and ectopic GFP-FOR20 in HeLa cells. GAPDH was used as an internal control. (**b** and **c**) Wound healing experiments showed the migration of cells with the indicated treatments. Dashed lines indicate the wound edges. Distance of the wound was measured by ImageJ software and data are presented as mean ± SD. Bar, 100 μm. (**d** and **e**) Transwell analysis exhibited the migration of the indicated cells. Quantitative

data of randomly selected fields (n > 3) are shown. Bar, 20 μm. *$P$ < 0.05 and **$P$ < 0.01, one-way ANOVA.

**Supplementary Movie S1. *In vitro* microtubule dynamics assay in the absence of FOR20**, related to Figure 3. The dynamic microtubules were polymerized with 12 μM Alexa 488-labeled tubulin (green) by extension of GMPCPP-stabilized TAMRA-labeled microtubule seeds (red), and recorded by TIRF microscopy.

**Supplementary Movie S2. *In vitro* microtubule dynamics assay with 0.1 μM FOR20**, related to Figure 3. The dynamic microtubules were polymerized with 12 μM Alexa 488-labeled tubulin (green) by extension of GMPCPP-stabilized TAMRA-labeled microtubule seeds (red) in the presence of 0.1 μM FOR20, and imaged by TIRF microscopy.

**Supplementary Movie S3. *In vitro* microtubule dynamics assay with 1 μM FOR20**, related to Figure 3. The dynamic microtubules were polymerized with 12 μM Alexa 488-labeled tubulin (green) by extension of GMPCPP-stabilized TAMRA-labeled microtubule seeds (red) with 1 μM FOR20, and imaged by TIRF microscopy.

**Supplementary Movie S4. *In vitro* microtubule dynamics assay with GFP-EB1**, related to Figure 5b. The dynamic microtubules were polymerized with 12 μM Alexa

594-labeled tubulin by extension of GMPCPP-stabilized TAMRA-labeled microtubule seeds (red) in the presence of 30 nM GFP-EB1, and imaged by TIRF microscopy.

**Supplementary Movie S5. *In vitro* microtubule dynamics assay with GFP-FOR20**, related to Figure 5c. The dynamic microtubules were polymerized with 12 μM Alexa 594-labeled tubulin by extension of GMPCPP-stabilized TAMRA-labeled microtubule seeds (red) in the presence of 150 nM GFP-FOR20, and recorded by TIRF microscopy.

**Supplementary Movie S6. *In vitro* microtubule dynamics assay in the absence of GFP-FOR20**, related to Figure S1. The dynamic microtubules were polymerized with 12 μM Alexa 594-labeled tubulin by extension of GMPCPP-stabilized TAMRA-labeled microtubule seeds (red), and recorded by TIRF microscopy.

**Supplementary Movie S7. *In vitro* microtubule dynamics assay with 1 μM GFP-FOR20**, related to Figure S1. The dynamic microtubules were polymerized with 12 μM Alexa 594-labeled tubulin by extension of GMPCPP-stabilized TAMRA-labeled microtubule seeds (red) in the presence of 1 μM GFP-FOR20, and imaged by TIRF microscopy.

**Supplementary Movie S8. The migration of control cells at the wound edge**,

related to Figure 7d. HeLa cells were transfected with pBS/U6 vector for 72 h and subjected to wound healing assays. The migration of cells at the wound edge were recorded by video microscopy.

**Supplementary Movie S9. The migration of FOR20-depleted cells at the wound edge**, related to Figure 7d. HeLa cells were transfected with pBS/U6-FOR20 plasmid for 72 h and processed for wound healing assays. The migration of cells at the wound edge were imaged by video microscopy.

**Supplementary Movie S10. The random migration of control cells**, related to Figure 7g. The migration of HeLa cells transfected with pBS/U6 vector for 72 h were recorded by video microscopy.

**Supplementary Movie S11. The random migration of FOR20-depleted cells**, related to Figure 7g. The migration of HeLa cells transfected with pBS/U6-FOR20 plasmid for 72 h were imaged by video microscopy.
